# Idiopathic Fascicular Ventricular Tachycardia in a Pediatric Patient With Suicidal Ideation and Self-Injurious Behavior

**DOI:** 10.7759/cureus.50061

**Published:** 2023-12-06

**Authors:** Margaret A Uchefuna, Ruth Y Eletta, Arleen Delgado, Susan Beker

**Affiliations:** 1 Department of Pediatrics, Woodhull Medical Center, New York, USA; 2 Department of Pediatric Cardiology, New York University, New York, USA

**Keywords:** idiopathic, self-harm, suicidal ideations, child and adolescent, fascicular ventricular tachycardia, ventricular tachycardia (vt)

## Abstract

Idiopathic fascicular ventricular tachycardia (IFVT) is an arrhythmia that occurs in a structurally normal heart and may present with sudden onset in a healthy individual. We present the case of a 10-year-old female child, with no pertinent medical history, who complained of palpitations and shortness of breath, which was followed by suicidal ideations. On presentation to the ER, tachycardia was noted with other vital signs within normal limits. Labs were unremarkable. EKG showed wide-complex tachycardia with right bundle branch block and left superior axis, consistent with idiopathic left ventricular fascicular tachycardia. The echocardiogram showed normal cardiac structure. She was transferred to the cardiovascular care unit and intravenous verapamil was given with the resolution of symptoms and reversal of tachycardia. She remained hemodynamically stable and was subsequently discharged on oral verapamil. This case report is aimed at raising awareness of the different ways IFVT can manifest, aiding physicians to easily recognize the zebra among the horses.

## Introduction

Ventricular tachyarrhythmias are arrhythmias characterized by a fast heart rate; 70-90% occur in the right ventricle, with or without structural defects. They can be classified based on anatomical location, clinical features, duration and the presence or absence of cardiac disease [1]. 

Idiopathic fascicular ventricular tachycardia (IFVT) arises in the absence of structural disease of the heart. It can occur acutely in a healthy person and affects the left ventricle less than 15% of the time [1]. An electrocardiogram is very helpful in diagnosis and although IFVT can be confused with supraventricular tachycardia with aberrancy, since both can present with wide-complex tachycardia and right bundle branch block, certain findings can help differentiate them [2,3].

The first-line treatment of IFVT is verapamil, with a positive response seen in more than 80% of patients [4]. In verapamil-resistant IFVT, catheter ablation can be done with an efficacy yield greater than 85% [5,6]. The outcome of IFVT is variable and can range from complete resolution to progression that can lead to cardiomyopathy and, sometimes, death [1]. 

We present the case of a pre-adolescent female child with an unremarkable medical history, who presented with intra-fascicular verapamil-sensitive ventricular tachycardia.

## Case presentation

A 10-year-old female child, with no pertinent medical history, was presented to the emergency room (ER) by her mother with complaints of suicidal ideation associated with self-injurious behavior. The child had witnessed a scenario of bullying in school a day prior to the current presentation. On returning home from school on the day of the presentation, she reported palpitations and shortness of breath, which her mom initially attributed to anxiety. However, this was followed by the onset of suicidal ideations, and her mom brought her to the ER. It was noted that the patient had scratched herself with a pair of scissors on two occasions in the preceding two weeks because she was emotionally overwhelmed by events and strained relationships at home and in school.

**Figure 1:**
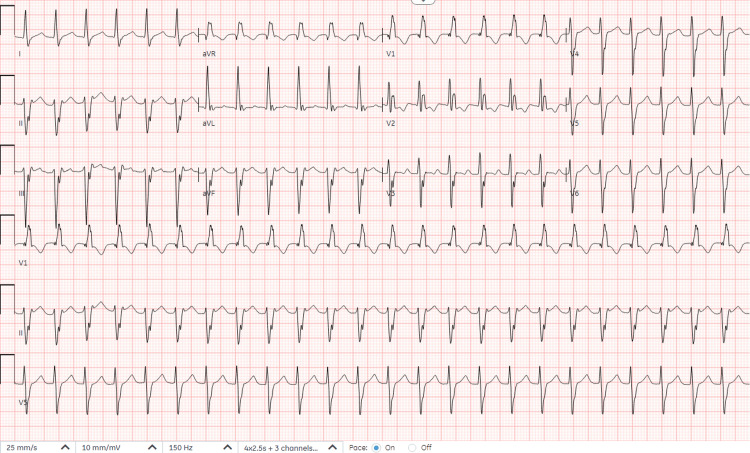
Left axis deviation (R axis: -63 degrees, T axis: 36 degrees), right bundle branch block with taller "left rabbit ears", R/S ratio less than one in V6 and wide QRS tachycardia (heart rate: 145 beats per minute) with QRS duration: 130 ms, QT interval: 352 ms and QTc: 546 ms.

At the current presentation in the ER, tachycardia was noted, with a heart rate of 145 beats/minute while other vital signs were within normal limits. The tachycardia persisted despite hydration. Labs were unremarkable, while EKG showed left axis deviation (R axis: -63 degrees, T axis: 36 degrees), right bundle branch block with taller "left rabbit ears" in V1 and V2, R/S ratio less than one in V6 and wide QRS tachycardia (heart rate: 145 beats per minute) with QRS duration: 130 milliseconds, QT interval: 352 milliseconds and QTc: 546 milliseconds as seen in Figure 1, consistent with idiopathic left ventricular fascicular tachycardia.

**Figure 2:**
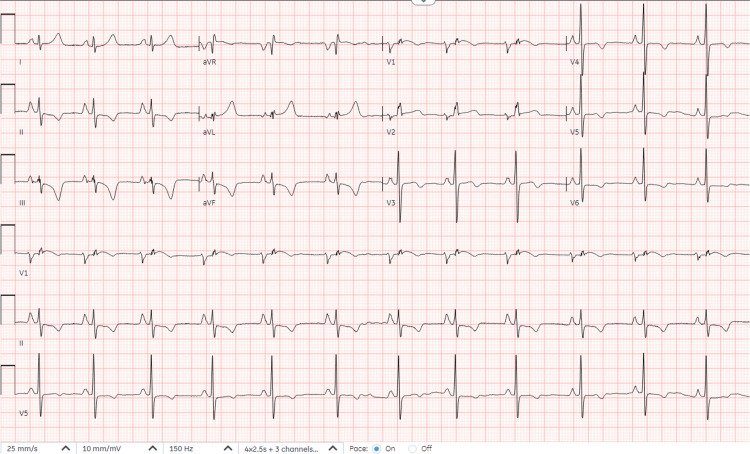
Normal sinus rhythm, left axis deviation (P axis: 53 degrees, R axis: -30 degrees, T axis: -53 degrees), and resolution of wide complex tachycardia (heart rate now 73 beats per minute) with QRS duration: 78 ms, PR interval: 132 ms, QT interval: 380 ms and QTc: 418 ms, after administration of verapamil.

The echocardiogram showed normal cardiac structure. A consultation to the psychiatry team was done and the patient was cleared from a psychiatric perspective. She was transferred to the cardiovascular care unit and was given intravenous verapamil with resolution of symptoms and reversal of the EKG to normal sinus rhythm with left axis deviation (P axis: 53 degrees, R axis: -30 degrees, T axis: -53 degrees), heart rate: 73 beats per minute, QRS duration: 78 milliseconds, PR interval: 132 milliseconds, QT: 380 milliseconds and QTc: 418 milliseconds as shown in Figure 2.

She remained hemodynamically stable, and oral verapamil eventually commenced with titration of the dose till her heart rate was within normal limits. She was discharged on oral verapamil 120 mg daily.

## Discussion

Ventricular tachycardia involving the fascicular system can occur in the presence or absence of structural heart disease. Posterior fascicular ventricular tachycardia is the most common form of IFVT, and is seen in about 90% of cases [2]. It usually occurs in older adolescents and adults, with a male predominance of 60-80% [2]. Our patient, however, was a pre-adolescent female.

IFVT can occur with or without exertion and can also be triggered by extremes of emotions [2], as was probably the case in our patient who was emotionally overwhelmed and developed it after witnessing a bullying episode in school. A patient with IFVT can present with symptoms of dizziness, palpitations, and fainting or near-fainting. On EKG, features of IFVT may consist of wide complex tachycardia, right bundle branch block (RBBB), superior left axis deviation (usually if posterior fascicle is affected) or right axis deviation (if anterior fascicle is involved) [2].

Early identification and diagnosis are essential because a delayed diagnosis can result in cardiac dysfunction. The closest differential of our patient's diagnosis of IFVT was supraventricular tachycardia (SVT) with aberrancy because although ventricular tachycardia (VT) is commoner in adults and SVT in younger patients, both can present in the absence of structural heart disease and with EKG findings of wide QRS tachycardia and right-bundle branch block [7].

The Brugada algorithm is the most widely known approach for distinguishing VT from SVT with aberrancy [7]. Four questions are addressed in a stepwise fashion: absence of RS complex in all precordial leads, longest RS interval greater than 100 ms in any precordial lead, presence of AV dissociation, and presence of morphological criteria for VT in leads V1-2 and V6. An affirmative response to any of the questions when applied in a systematic manner, has been found to have a sensitivity of 98.7% and specificity of 96.5% in diagnosing VT [3].

Our patient's EKG was negative for the first three criteria, so we used the morphological criteria for VT. The QRS polarity was predominantly positive in precordial leads V1 and V2 (RBBB morphology) and, in the presence of a left axis deviation, this suggests VT [7]. The double peaking of the R wave seen in V1 and V2 is referred to as "rabbit ears". When the left rabbit ear is taller than the right in V1 (notched downslope of the R wave) or V2, as seen in this case, the diagnosis of VT is more favored [7]. In addition, the presence of a small R wave and deep S wave in lead V6, resulting in an R/S ratio <1 is more in keeping with VT [7].

The use of verapamil can halt tachycardia and reduce the number of recurrent episodes, as described by Belhassen et al. [8]. The greatest efficacy with the use of verapamil is seen in people whose symptoms are mild. When symptoms are moderate to severe or persistent, catheter ablation becomes necessary [9]. In view of treatment implications, distinguishing IFVT from other forms of ventricular tachycardia is imperative as medications like adenosine are ineffective in the treatment of IFVT. Similarly, verapamil given in the setting of other forms of ventricular tachycardia can be fatal [10].

## Conclusions

IFVT is a medical emergency. Clinical presentation is variable. The palpitations that occur in IFVT can trigger anxiety with suicidal ideations and a feeling of impending doom, causing the underlying heart problem to be camouflaged as just another adolescent with psychiatric issues. 

A confirmatory response to any of the four criteria using the stepwise Brugada algorithm and a lack of response to adenosine should prompt a high index of suspicion for IFVT. Prompt intervention is key to improved outcomes and aversion of sudden cardiac death. This case report is aimed at raising awareness of the different ways IFVT manifests, aiding physicians to easily recognize the zebra among the horses.
